# TSLP is involved in expansion of early thymocyte progenitors

**DOI:** 10.1186/1471-2172-8-11

**Published:** 2007-07-18

**Authors:** Qi Jiang, V McNeil Coffield, Motonari Kondo, Lishan Su

**Affiliations:** 1 Lineberger Comprehensive Cancer Center, School of Medicine, UNC at Chapel Hill, Chapel Hill, NC 27599, USA; 2 Department of Microbiology and Immunology, School of Medicine, UNC at Chapel Hill, Chapel Hill, NC 27599, USA; 3 Curriculum in Genetics and Molecular Biology, School of Medicine, UNC at Chapel Hill, Chapel Hill, NC 27599, USA; 4 Department of Immunology, Duke University Medical Center, Durham, NC 27710, USA

## Abstract

**Background:**

Thymic stromal derived lymphopoietin (TSLP) is preferentially and highly expressed in the thymus, but its function in T cell development is not clear.

**Results:**

We report here that TSLP, independently or in combination with IL-7, enhances thymopoiesis in the murine fetal thymic organ culture (FTOC) model. Furthermore, TSLP preferentially increases the number and proliferation of the (DN1 and DN2) pro-T progenitor cells, and FTOC lobes from TSLP receptor-null mice show a decreased number of these cells. Finally, DN1-DN2 cells expanded with TSLP in vitro are functional T progenitors that are able to differentiate into mature T cells in fetal or adult thymus organs.

**Conclusion:**

Together, these data suggest that TSLP plays an important role in expansion of thymocyte progenitors and may be of value for expanding T progenitor cells in vitro.

## Background

T cell development in the thymus is characterized by a series of distinct steps marked by changes in the expression of cell surface proteins [1]. Hematopoietic stem cells (HSC) from the fetal liver during early gestation or the bone marrow later in development, home to the thymus to begin the process of commitment and maturation to the T cell lineage [2,3]. The first critical checkpoint occurs in very early CD4^-^CD8^- ^double-negative (DN) thymocyte progenitors, which also lack surface expression of the T cell receptor (TCR) and are further subdivided into four stages (DN1-4) based on their surface expression of CD44 and CD25. Successful rearrangement of the genes encoding the TCRβ chain in DN3 cells results in the expression of a pre-TCR on the cell surface. Subsequent signaling through the pre-TCR and growth factor receptors induces cell proliferation and differentiation. The cells mature into double positive (DP) thymocytes that express both CD4 and CD8 coreceptors as well as the mature αβ TCR on the cell surface. A program, termed positive selection, initiated by TCR-mediated recognition of complexes of self-peptides and major histocompatibility complex (MHC) proteins occurs in a minority of DP thymocytes are further differentiated during the process of positive and negative selection, resulting in the production of mature CD4 and CD8 lineage T cells.

T cell development in the thymus is tightly regulated by thymic microenvironment composed of cytokine-producing stromal cells. IL-7 produced from thymic stromal cells plays a critical role in the development of T cells, as mice lacking IL-7 (*IL7*^-/-^) display a marked reduction in thymic cellularity [4,5]. Deficiency in IL-7Rα (*IL7Rα *^-/-^) seems to cause a phenotype similar, but more severe, to that seen in the absence of IL-7, exhibiting more severely reduced thymic cellularity and defective T cell maturation [6]. Therefore, IL7-independent signaling pathway via IL-7Rα is implicated in T cell development.

The IL-7 receptor consists of the common γ chain (γc) and IL-7Rα. The receptor complex for TSLP, an IL7-like cytokine, also consists of the IL-7Rα chain and the TSLP receptor (TSLPR) [7]. Like the γC-mediated signal transduction, signaling through TSLPR also activates the STAT-5 protein, but independently of the activation of Janus kinase-3 [8]. Thus, IL-7Rα can signal by TSLP in the absence of IL-7 and, in the absence of IL-7Rα, both TSLP and IL-7 signals are blocked.

TSLP is a type 1 cytokine which was originally identified as a growth factor in the supernatant of a thymic stromal cell line [9]. However, its effect on thymopoiesis has not been clearly demonstrated yet. It has been reported that TSLP promotes the proliferation and differentiation of B-cell progenitors from fetal liver [10,11]. TSLP can replace IL-7 in supporting the maturation of B-cells from pro-B precursors and B cell progenitors fail to develop from these B cell precursors if no TSLP or IL-7 is present. In transgenic mice, ectopic expression of TSLP in mice causes imbalances in lymphopoiesis and myelopoiesis [12].

It has been reported that human TSLP enhances the maturation of CD11c^+ ^dendritic cells to modulate functional differentiation of CD8^+ ^cells and to support proliferation of naïve T cells [13,14]. TSLP is also an important factor involved in allergic airway inflammation [15,16]. Although human TSLP does not directly interact with human T cells, TSLP is produced by Hassall's corpuscles in the human thymus, where it instructs thymic dendritic cells to convert high affinity self-reactive T cells into CD4^+^CD25^+^Foxp3^+ ^regulatory T cells [17]. In TSLP receptor knock-out mice, normal development of T or B cells is reported [18]. Murine TSLP preferentially enhances the expansion and survival of CD4^+ ^T cells both in vitro and in vivo, especially in the absence of IL7-mediated signaling as TSLPR/γc double knockout mice have a greater lymphoid defect than γc single KO mice [19]. In murine neonatal thymus, TSLP produced from medullary thymic epithelia cells (mTEC) contributes to the expression of FoxP3 and the maturation of natural regulatory T cells [20].

We demonstrate here that murine TSLP also plays a role in regulating early T cell development. In the murine fetal thymus organ culture (FTOC) model, TSLP alone, or in combination with IL-7, enhances T cell development. TSLP preferentially increases the number and proliferation of the pro-T cells (DN1 and DN2). Furthermore, the DN1-DN2 cells expanded with TSLP in vitro are functional T progenitor cells that are able to differentiate into mature T cells in FTOC ex vivo or when injected into mice in vivo. Together, these data suggest that TSLP is involved in development and expansion of thymocyte progenitors.

## Results

### TSLP and IL-7 both enhance thymopoiesis in fetal thymic organ culture (FTOC)

**Figure 1:**
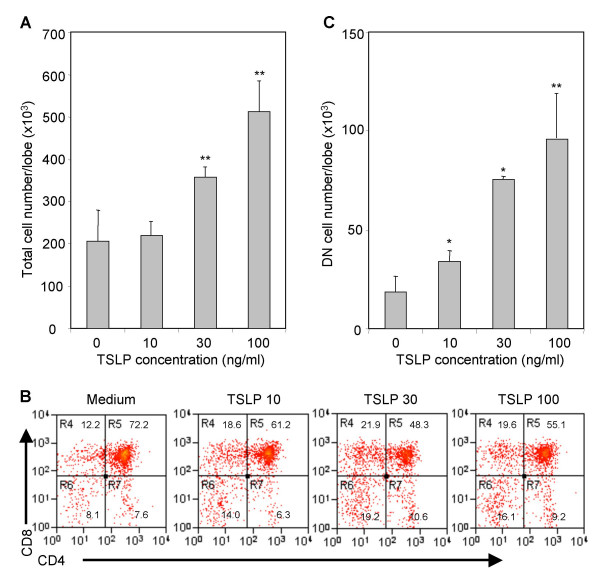
TSLP enhances early thymopoiesis in mouse fetal thymic organ culture. E15 fetal thymus organs were cultured with different doses of TSLP (10, 30 or 100 ng/ml) (A) for 2 weeks. Total thymocytes were harvested and counted. B, TSLP increased the relative frequencies of CD4^-^CD8^- ^(DN) and CD4^-^CD8^+ ^thymocytes in FTOC. C, TSLP preferentially increased the number of DN thymocyte cells. Shown are representative data from at least 4 independent experiments. Standard deviation (SD) is shown as error bars. *: P < 0.05, * *: P < 0.01, compared with medium control.

Because TSLP and IL-7 share the IL-7 receptor alpha (IL-7Rα) chain, and IL-7Rα-null mice show more dramatic defects in thymopoiesis than IL7-null mice, we investigated the importance of TSLP in thymopoiesis. Increasing doses of TSLP were added to FTOC. Both high doses (100 ng/ml and 30 ng/ml) resulted in a significant increase (2.5 times and 1.8 times, respectively, compared with control) in total cell number (Fig. 1A and Table 1). When the thymocyte subsets were analyzed, we detected a preferential increase of DN and SP8 thymocytes, and a relative decrease in CD4^+^CD8^+ ^DP cells (Fig. 1B). At higher doses of TSLP, there was a significant increase in total cell numbers of all thymocyte subsets including CD4^-^CD8^- ^(DN), CD4^+^CD8^+ ^(DP), CD4^+^CD8^- ^(SP4) and CD4^-^CD8^+ ^(SP8) thymocytes in TSLP treated FTOCs (Table 1). Interestingly, the DN population was increased even with 10 ng/ml of TSLP, a concentration at which there was no significant increase in total thymocytes (Fig. 1C). Therefore, the DN cells seem to respond more efficiently to TSLP than DP and SP thymocytes during thymopoiesis.

**Table 1:** Effect of TSLP on thymocyte subpopulations in FTOC

Cell # (×10^3^)^a^	Medium	TSLP 10 ng/ml	TSLP 30 ng/ml	TSLP 100 ng/ml
Total	205.7 +/- 73.4^b^	220 +/- 32.5	356.7 +/- 21.9*	513.3 +/- 72.5**
	(1×)^c^	(1×)	(1.7×)	(2.5×)
CD4-CD8+	31.0 +/- 9.5	47.3 +/- 8.7	72.3 +/- 15.9*	103.7 +/- 16.9**
	(1×)	(1.5×)	(2.3×)	(3.3×)
CD4+CD8+	138.5 +/- 51.3	124.3 +/- 18.3	169.4 +/- 5.6	260.9 +/- 17.6*
	(1×)	(1×)	(1.2×)	(1.9×)
CD4-CD8-	18.7 +/- 8.0	34.1 +/- 5.4*	75.7 +/- 1.2**	96.2 +/- 23.0**
	(1×)	**(1.8×)**	(4.0×)	(5.1×)
CD4+CD8-	17.5 +/- 6.9	14.3 +/- 1.3	39.3 +/- 0.4**	52.3 +/- 18.6*
	(1×)	(1×)	(2.2×)	(3.0×)
DN1^d^	0.6 +/- 0.2	1.6 +/- 0.3*	3.0 +/- 0.5**	5.7 +/- 0.9**
	(1×)	**(2.7×)**	(5.0×)	(9.6×)
DN2	1.9 +/- 1.0	6.4 +/- 1.8*	9.6 +/- 5.3**	19.4 +/- 2.5**
	(1×)	**(3.4×)**	(5.1×)	(10.3×)
DN3	10.6 +/- 5.0	12.0 +/- 3.4	31.2 +/- 8.3	26.7 +/- 16.5
	(1×)	(1×)	(2.9×)	(2.5×)
DN4	0.9 +/- 0.4	2.0 +/- 0.4*	6.5 +/- 1.1**	5.5 +/- 1.5**
	(1×)	**(2.2×)**	(6.9×)	(5.9×)

^a ^E15 FTOC with different doses of TSLP were performed for 2 weeks. Thymocytes were harvested and analyzed by FACS. The number (×10^3^) of thymocyte subsets recovered from each FTOC lobe is shown. The data is a summary of 4 independent experiments.

^b ^Average number +/- SD;

^c ^Fold over medium control.

^d ^Thymocytes that were negative for expression of CD3/CD4/CD8 were analyzed for DN1 to DN4 subpopulations based on the expression of CD44 and CD25. * p < 0.05; ** p < 0.01, compared with medium control.

**Figure 2:**
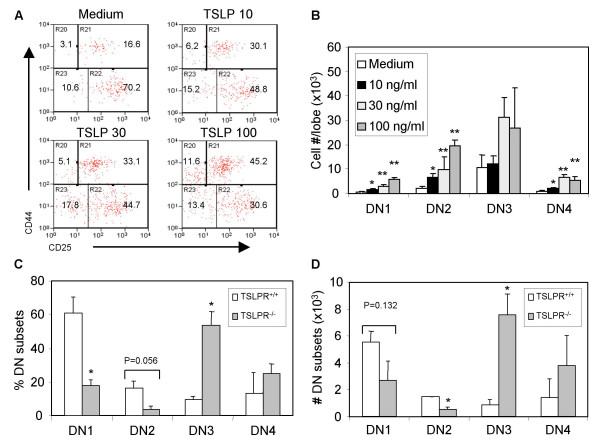
DN1 and DN2 cells are preferentially increased by TSLP in FTOC and decreased in TSLPR^-/- ^FTOC. A and B, FTOCs using WT E15 thymic lobes were performed with different doses of TSLP (10, 30 or 100 ng/ml) for 2 weeks. Thymocytes that were negative for expression of CD3/CD4/CD8 were further analyzed for DN subpopulation based on the expression of CD44 and CD25. Shown are representative data from at least 4 independent experiments. SD is shown as error bars. *: P < 0.05, * *: P < 0.01, compared with medium control. C and D, FTOCs from littermate TSLPR^+/+ ^or TSLPR^-/-^embryos were cultured for 2 weeks and DN cells were analyzed. The data is a summary of 3 independent experiments. Error bars indicate SD, * P < 0.05, * *: P < 0.01, compared with TSLPR^+/+ ^control lobes.

When the CD3^-^CD4^-^CD8^- ^(DN) thymocytes were analyzed for DN1-DN4 subsets, TSLP enhanced the frequencies and total number of DN1 (CD44^+^CD25^-^), DN2 (CD44^+^CD25^+^), and DN4 (CD44^-^CD25^-^) thymocytes (Fig. 2A, B and Table 1). Again, even low doses of TSLP (10 ng/ml) appeared to preferentially expand the DN1, DN2 and DN4 pro-/pre-T cells. When the TSLPR^-/- ^FTOC was performed in the presence of recombinant TSLP, neither total cell numbers nor the numbers of different DN subsets changed compared with medium control (data not shown), demonstrating the specificity of TSLP on thymopoiesis.

To further demonstrate that TSLP receptor mediated signaling is important for early thymopoiesis, we analyzed FTOC fetal thymus lobes from TSLPR^-/- ^mice. Compared with littermate TSLPR^+/+ ^thymic lobes, the percentages and total numbers of DN1 and DN2 were reduced (Fig. 2C and 2D). Notably, the DN3 subset was increased in TSLPR^-/- ^thymic lobes when compared with wild type control, probably due to compensatory IL-7 signaling. These results demonstrate an important role for TSLP in thymopoiesis at the early DN stage, which is dispensable during mouse development and adaptation.

**Figure 3:**
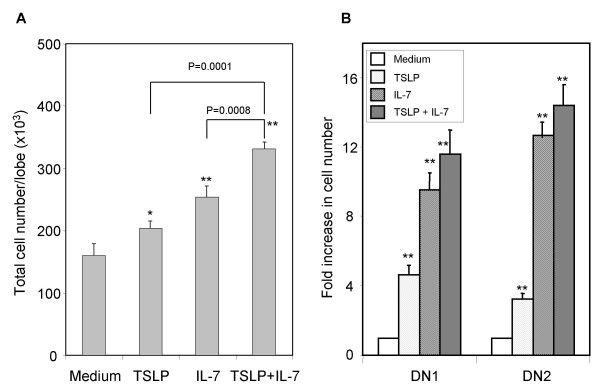
TSLP and IL-7 both enhances early T progenitor cells and thymopoiesis in mouse fetal thymic organ culture. E15 fetal thymus organs were cultured with either TSLP (30 ng/ml) and/or IL-7 (100 ng/ml) for 2 weeks. Total thymocytes were harvested and counted (A). Thymocytes that were negative for expression of CD3/CD4/CD8 were further analyzed for DN subpopulation based on the expression of CD44 and CD25 (B). The data is a summary of 2 independent experiments. Standard deviation (SD) is shown as error bars. *: P < 0.05, * *: P < 0.01, compared with medium control.

We also analyzed the activity of TSLP with and without IL7 in FTOC. Either TSLP or IL-7 in FTOC resulted in significantly increased numbers of total thymocytes (Fig. 3A). When the effect of IL-7 on early DN thymocyte progenitors in FTOC was analyzed (Fig. 3B), IL-7 also increased the number of DN1 and DN2 cells. IL-7 and TSLP increased DN1 cells by 9.6 and 4.7 fold, respectively; and DN2 cells by13.7 and 3.2 fold respectively. The combination of IL-7 and TSLP increased the numbers of DN1 and DN2 cells when compared with IL-7 alone. These data suggest that both TSLP and IL-7 can promote the expansion of early thymocytes.

### TSLP stimulated proliferation of DN1 and DN2 thymocytes in FTOC

**Figure 4:**
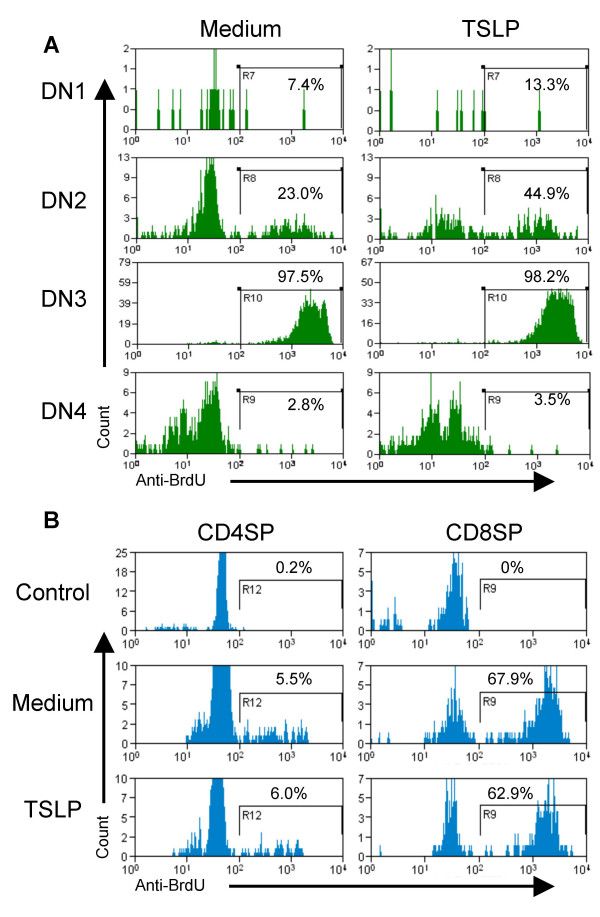
TSLP increases proliferation of DN1 and DN2 thymocytes in FTOC. E17 FTOCs were cultured with or without TSLP (30 ng/ml) for 48 hours, and BrdU was added for the last 4 hours before analysis. (A) Thymocytes from each FTOC lobe were harvested, stained for CD44, CD25, CD3, CD4, CD8 and BrdU. Thymocytes that were negative for expression of CD3, CD4 and CD8 were analyzed for DN subpopulations and relative BrdU incorporation. (B) The same thymocytes were analyzed for BrdU incorporation in CD3^+^CD4^+ ^or CD3^+^CD8^+ ^cells. The data is representative of two independent experiments. *, indicates p < 0.05; **, p < 0.01.

The enhancement in the number of DN cells from fetal thymus by TSLP led us to investigate whether TSLP is important for the proliferation of these thymocyte subsets. Because the levels of DN cells recoverable from E15 FTOC were too low for the BrdU-labeling assay, we employed E17 fetal thymic lobes to investigate the proliferation response of DN cells stimulated with TSLP. We found that the percentage and number of BrdU^+ ^DN1 and DN2 cells stimulated with TSLP were approximately doubled compared to control (Fig. 4A). The proliferation of DN4 subset, but not DN3, also was slightly enhanced by TSLP. In contrast, TSLP did not change the BrdU^+^percentages of CD4SP and CD8SP cells in FTOC (Fig. 4B). These data suggest that TSLP specifically increases the proliferation of early DN thymocyte progenitors.

### DN1-DN2 cells expanded by TSLP still have T progenitor activity to generate phenotypically mature thymocytes

**Figure 5:**
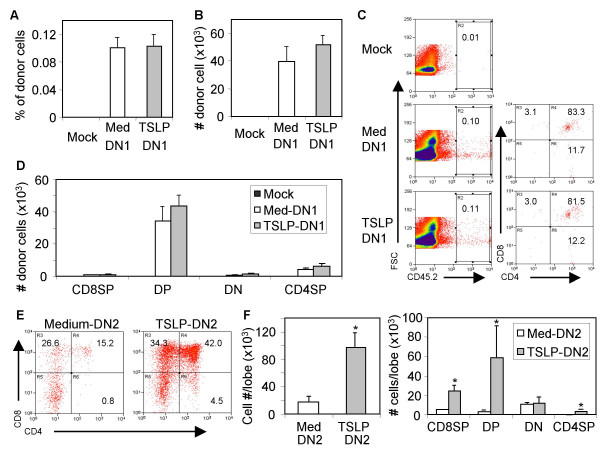
DN1 and DN2 cells from TSLP-treated FTOC have T progenitor activity to generate phenotypically mature thymocytes. DN1 and DN2 cells from FTOC lobes (Ly5.2) cultured with TSLP (30 ng/ml) or medium control were purified. Sorted DN1 cells (4 × 10^3^/mouse) were injected directly into thymi of irradiated (500 rad) Ly5.1 recipient mice with fresh carrier cells (Ly5.1^+ ^thymocytes, 1 × 10^5^/mouse). Two weeks after injection, thymocytes from recipient mice were analyzed for % donor cells (A), total number donor cells in the thymus (B), and % and number (C and D) of different donor thymocyte subsets. E and F, Sorted DN2 cells (1 × 10^3^/well) were seeded into 2-dG treated E15 thymic lobes by hanging drop culture, and cultured as FTOC for 2 weeks. Thymocytes were harvested and analyzed as above. The data is representative of two independent experiments. Bars indicate SD, * P < 0.05, compared with medium control.

It is not clear if these DN cells treated and expanded by TSLP in FTOC in vitro are compromised in their T progenitor activity. We assessed the potential of DN1 and DN2 progenitors from TSLP-treated FTOC both in vivo in adult thymus and in vitro in reconstituted FTOC. Ly5.2^+ ^DN1 thymocyte progenitors sorted from FTOC cultured with or without TSLP were intrathymically injected into irradiated Ly5.1^+ ^recipient mice. Two weeks after injection, cells from thymus were harvested and analyzed. DN1 cells from mock or TSLP treated FTOC were similar in generating donor-derived (Ly5.2^+^) immature or mature thymocytes in mice (Fig. 5A and 5B). Thus, DN1 cells from TSLP-treated FTOC had the same thymocyte differentiation capacity as control DN1 cells in adult thymus in vivo, as determined by CD4 and CD8 expression of donor-derived thymocytes (Fig. 5C and 5D).

In reconstituted FTOC assay using 2-dG treated E15 thymic lobes, DN2 cells from TSLP-treated FTOC in vitro also efficiently differentiated into DP and mature SP4^+ ^and SP8^+ ^thymocytes (Fig. 5E). Total cell number and numbers of SP4, DP and SP8 cells recovered from FTOC lobes repopulated with TSLP-treated DN2 cells were at least as high (or higher) as control DN2 cells (Fig. 5F). These data indicate that DN1 and DN2 cells treated and expanded in FTOC with TSLP in vitro still possess T progenitor activity. Together, our data suggest that TSLP plays an important role in expansion of thymocyte progenitors and may be of value to expand T progenitor cells in vitro.

## Discussion

TSLP was initially reported as a cytokine secreted by a thymic stromal cell line that could replace IL-7 in supporting B lymphocyte development [11] and B cell maturation [9]. It has since been shown to also affect myeloid lineage cells in both mice and humans. Human TSLP produced by epithelial cells and keratinocytes, via its interaction with dendritic cells, has emerged as an important trigger of allergic T-cell responses and of CD4^+ ^T cell homeostatic expansion [13,14,21]. In addition, TSLP has been recently reported to modulate mature CD4^+ ^T cell proliferation and survival [19]. However, Sims et al. [22] have reported that addition of TSLP to purified CD4^-^8^- ^DN thymocytes in vitro results in no change in cell proliferation. This may be due to lack of other co-factors that are present in the FTOC culture. Interestingly, when combined with IL-1, TSLP can lead to a significant expansion of these purified DN cells in vitro. We report here that TSLP enhances thymopoiesis, and preferentially increases the number and proliferation of the pro-/pre-T (DN1, DN2 and DN4) cells in FTOC assays. In addition to increasing the number of thymocytes (Table 1), TSLP may also promote differentiation of thymocytes as TSLP increased both the numbers and percentages of CD8SP and CD4SP cells (Fig. 1B) but not their proliferation (Fig. 4B). Therefore, TSLP appears to affect myeloid cells, B cells, and T cells at various stages of development.

It is of interest to note that IL-7 and TSLP play similar but non-overlapping roles in thymopoiesis. While IL-7 uses the IL7Rα/γc receptor complex, TSLP signals via a receptor comprising the IL-7Rα and a distinctive subunit, TSLPR [7,23]. IL-7 has been shown to support the growth of DN and CD4^-^CD8^+ ^subpopulations in FTOC system [24], but to inhibit the production of total CD4^+^CD8^+ ^DP cells. IL-4, another cytokine which uses the common cytokine receptor γc [25], can also block the development of CD4^+^CD8^+ ^thymocytes in FTOC [26]. In our study, TSLP increases the cell number of all subpopulations (Table 1), but preferentially enhances proliferation of DN1, DN2, and DN4 cells (Fig. 3). Based on the expression of CD24 and CD117, DN1 cells can be further subdivided into 5 subsets [27]. TSLP may affect the proliferation and/or differentiation of some or all these subsets. Unfortunately, we have been unable to obtain enough cells for a thorough analysis of the responsiveness of these DN1 subsets to TSLP in FTOC. These data suggest that TSLP enhances T progenitor cell expansion and supports their succeeding thymocyte differentiation pathway.

TSLPR-deficient adult mice have shown no significant developmental abnormality in T cells and B cells [18,19]. Studies in TSLPR/γc DKO mice have indicated a role for TSLP in CD4^+ ^T cell homeostasis [19]. Similarly, TSLP is implicated in the induction of splenic B cells from studies with TSLPR/*Jak3*^-/- ^DKO mice [18]. These findings suggest the TSLP has an overlapping role with IL-7 (and possibly other cytokines) in controlling lymphopoiesis. Interestingly, TSLP seems to promote B lymphocyte production from fetal liver-, but not adult BM-, derived pro-B cells [28]. The normal phenotype of T and B cells in TSLPR KO mice suggests that TSLP may be involved in fetal thymopoiesis, and other cytokines such as IL-7 may play a compensatory or adaptive role in T/B cell development during development of TSLPR-null mice [4,6,29].

## Methods

### Animals

B6/Ly5.1 and B6/Ly5.2 mice were purchased from the Jackson Laboratory and TSLP-R-deficient mice were provided by Dr. J. Ihle [18] and backcrossed to B6/Ly5.2 background for more than 4 generations. All mice were housed in a sterile animal facility at the University of North Carolina at Chapel Hill [30]. Mice were mated for 16 h and fetuses were removed from pregnant females at various days of gestation (plug date = day 0).

### Media and reagents

FTOC medium contains RPMI 1640 (GIBCO BRL) supplemented with L-glutamine, 10 % FBS, 50 μM 2-ME, 2 mM L-glutamine, 1 % nonessential amino acid (GIBCO BRL), 10 mM HEPES, 1 mM sodium pyruvate solution (GIBCO BRL), 100 U/ml Penicillin and 100 mg/ml Streptomycin. 2-deoxyguanosine (2-dG) was purchased from Sigma-Aldrich (St. Louis, MO). Recombinant mouse TSLP was obtained from R&D Systems (Minneapolis, MN) and recombinant mouse IL-7 from PeproTech INC (Rocky Hill, NJ).

### Mouse fetal thymus organ culture (FTOC)

Thymic lobes from day 15 or day 17 embryos were cultured on the membranes directly with adding cytokines to the medium as described [31]. The lobes were cultured at 37°C in 5 % CO_2 _for indicated period, being re-fed with fresh FTOC medium plus cytokines every three days. For hanging drop-mediated transfer and organ culture of sorted cells, thymic lobes from day 15 embryos were cultured for 5 days in the presence of 1.35 mM 2-dG. The lobes were washed and co-cultured with sorted DN2 cells (1 × 10^3^/well) in hanging drops (40 μl/well) in Terasaki plates for 48 hours at 37°C. Lobes were rinsed with culture medium and organ culture on membranes for indicated period.

### FACS analysis

Cells isolated from fresh or cultured fetal thymic lobes were prepared as described. Monoclonal antibodies to CD3-ε, CD4, CD8, CD44, CD25, CD45.1, CD45.2, were obtained from BD Pharmingen (San Jose, CA), or Caltag (South San Francisco, CA).

BrdU analysis was performed by adding 25 μM BrdU (Sigma, St. Louis, MO) to the culture medium for 4 h before harvesting thymic lobes. Following extracellular staining, cells were fixed, permeabilized, and then stained with anti-BrdU-FITC (Becton Dickinson, Mountain View, CA). Events were collected on a FACScalibur (Becton Dickinson) and analysis was performed by using Summit v3.1 program [32].

### Intrathymic injection

Two to three months old recipient B6/Ly5.1 animals were administered with sterile pH2.0 water supplemented with neomycin sulfate. Sub-lethal irradiation of animals (500 rads) was administered with a Cesium source irradiator. The Institutional Animal Care and Use Committee approved all experiments. E15 thymic lobes were stimulated with TSLP (30 ng/ml) for 2 weeks, DN1 and DN2 cells were sorted by FACSVantage (Becton Dickinson). Sorted DN1 cells (4 × 10^3^/mouse) were injected into thymus of irradiated recipient mice with carrier cells (recipient type thymocytes: 1 × 10^5 ^cells/mouse). Two weeks after injection, mice were sacrificed and cells from thymus and spleen were harvested and analyzed by flow cytometry.

## Abbreviations

^1 ^TSLP, thymic stromal derived lymphopoietin

^2 ^FTOC, fetal thymus organ culture

## Authors' contributions

QJ carried out the whole studies and drafted the manuscript. VMNC carried out the part of fetal thymus organ culture. MK participated in the cell sorting and experimental design. LS conceived of the study, and participated in its design and coordination and helped to draft the manuscript. All authors read and approved the final manuscript.
